# Unraveling the Effects of Cold Stratification and Temperature on the Seed Germination of Invasive *Spartina alterniflora* Across Latitude

**DOI:** 10.3389/fpls.2022.911804

**Published:** 2022-06-29

**Authors:** Jingyang Cheng, Hao Huang, Wenwen Liu, Yupeng Zhou, Weipeng Han, Xiuyan Wang, Yihui Zhang

**Affiliations:** Key Laboratory of the Ministry of Education for Coastal and Wetland Ecosystems, College of the Environment and Ecology, Xiamen University, Xiamen, China

**Keywords:** invasive plants, cold stratification, seed germination, temperature, latitude, global warming

## Abstract

Seed germination is critical to the life history of plants, playing an important role in the successful recruitment, colonization, and even invasion of new individuals within and outside population distribution ranges. Cold stratification and temperature are the key factors affecting seed germination traits. Studying how these two factors drive geographical variation in seed germination is essential to analyze and predict the geographical distribution range of alien plants in novel habitats. *Spartina alterniflora*, native to the United States, was introduced into China in 1979 and has spread over 20° of latitude along the eastern coast of China. Germination plays a crucial role in *S. alterniflora*’s large-scale invasion and diffusion across latitude. To evaluate the effects of cold stratification and temperature on seed germination of *S. alterniflora* across latitude, we collected seeds at seven locations across latitude in China. We exposed these provenances to cold stratification at 4°C (0, 1, 3, and 5 months) and germination temperature (5°C, 15°C, 25°C, and 35°C) treatments in growth chambers. Seed germination was observed for 98 days, and we calculated germination rate, germination index, and germination time. Results indicated that longer cold stratification significantly promoted germination rate and germination index, but decreased germination time. Similarly, higher germination temperature significantly promoted germination rate and germination index, but decreased germination time. Moreover, there were significant interactive effects on germination traits between cold stratification and temperature. Seed germination traits showed linear relationships with latitude, indicating that *S*. *alterniflora* seeds from different provenances germinated at different times and adopted different germination strategies. The stratification and temperature are the most important factors regulating the dormancy and germination seeds, so they can be important drivers of this variation along latitude. Under scenarios of warmer regional temperature, seeds at higher latitudes could germinate earlier and have higher germination rate, which would favor a potential northern expansion of this invasive plant.

## Introduction

Seed germination is a critical stage in the life cycle of plants and plays a key role in the establishment, naturalization, and spread of their populations (Donohue et al., 2010). Several factors that affect germination (including cold stratification and germination temperature; Baskin and Baskin, 1998; Gillard et al., 2017) vary across latitude and thus drive the geographical distribution of plants (Brändle et al., 2003; Leiblein-Wild et al., 2014; Gillard et al., 2017). Understanding the importance of cold stratification time and germination temperature for seed germination characteristics is, therefore, key to analyzing and predicting the distribution and range of plant species (Cavieres and Arroyo 2000; Gremer et al., 2020), especially in the light of anticipated changes to local and regional climates (Walck et al., 2011; Zogas et al., 2020).

Dormancy ensures that seeds avoid the abiotic stresses associated with extreme cold and drought (Wagmann et al., 2012; Baskin and Baskin, 2014) and germinate under suitable conditions for subsequent growth (Bewley et al., 2013). In temperate and boreal regions, stratification helps break dormancy for seeds after the cold, wet, winter period (Baskin and Baskin, 2014). For example, cold stratification has been shown to increase germination percentage and germination rate in species such as *Arbutus unedo*, *Corylopsis coreana*, *Primula beesiana*, and *Sclerocarya birrea* (Moyo et al., 2009; Pipinis, 2017; Kim et al., 2018; Yang et al., 2020). Although longer stratification times are usually needed by species that experience longer winters (Zhang et al., 2019), prolonged seed stratification may reduce germination and subsequent seedling survival (Plyler and Carrick, 1993; Plyler and Proseus, 1996). Temperature also affects seed germination (Baskin and Baskin, 1977). For example, higher temperatures increased the germination rates of *Amaranthus tuberculatus*, *Setaria faberi*, and *Abutilon theophrasti* seeds (Leon et al., 2004; Chamorro et al., 2018). In many cases, a rise in temperature is a signal for seeds to germinate, and a drop in temperature is a signal to go dormant (Watt and Bloomberg, 2012).

These environmental factors (cold winter and warming spring) that drive stratification and germination temperature vary predictably by latitude, but recent studies of seed germination at different latitudes have shown contrasting results. Cross-species analyses have found that some species germinate faster at higher latitudes (Chamorro et al., 2018) and some slower (Zhou et al., 2021), suggesting species-specific responses to their environment. Yet, few studies have examined variation in seed germination within species over their geographic distribution in response to these factors (Baskin and Baskin, 1998; Molina-Montenegro et al., 2018; Solé-Medina et al., 2020).

An excellent system to study the relative importance of drivers of seed germination is the cordgrass *Spartina alterniflora* in China, introduced from the United States in 1979. The distribution now covers a range of over 20° (19°N–40°N) of latitude after 40 years of deliberate planting and natural dispersal (An et al., 2007; Zhang et al., 2017), which poses a serious threat to the Chinese coastal and wetland ecosystem (Li et al., 2009). Germination appears to play a crucial role in *S. alterniflora’*s large-scale latitude invasion and diffusion (Daehler and Strong, 1994; Liu et al., 2016), because stratification time and germination temperature vary throughout its range (Liu et al., 2021). *Spartina alterniflora* has an optimal cold stratification time of 4 months at 4°C (Biber and Caldwell, 2008). Longer or shorter periods of stratification reduce germination rates (Stalter, 1973; Seneca, 1974). Further, the expansion of *S. alterniflora* populations was closely related to local temperature (Loebl et al., 2006). The germination rate of *S. alterniflora* increased with increasing temperature from 5°C to 25°C (Yuan and Shi, 2009). Similarly, other traits such as germination characteristics (Liu and Zhang, 2021), vegetative growth (Liu et al., 2020a,b), flowering phenology (Somers and Grant, 1981; Crosby et al., 2015; Chen et al., 2021), seed set (Liu et al., 2016, 2017a), and life-cycle biomass allocation (Seneca, 1974; Liu et al., 2022) are also related to latitude in both invasive and native areas. However, the effects of stratification time and temperature gradient on germination characteristics have not been considered over a large-scale latitude gradient for *S. alterniflora*.

To evaluate the effects of cold stratification time and temperature on seed germination characteristics of populations from different latitudes, we sampled populations of *S. alterniflora* along a large-scale latitudinal gradient in coastal China. We exposed collected seeds from these different provenances to a range of cold stratification and temperature treatments and recorded their germination characteristics. The experiments addressed the following three questions: (1) What are the relative effects of cold stratification and temperature on seed germination? (2) How does seed germination vary along latitudinal gradients? (3) How do the effects of cold stratification and temperature on germination vary based on latitude?

## Materials and Methods

### Study Species

*Spartina alterniflora*, native to North America, is self-incompatible and wind-pollinated. Since its introduction into China in 1979, this species is now widely distributed along China’s coastline from 19° N to 40° N latitude (Zhang et al., 2017). *Spartina alterniflora* expands mainly through clonal growth and sexually reproducing seeds and maintains established populations mainly through clonal growth and sexual reproduction, and sexual reproduction is the main way of long-distance transmission (Daehler and Strong, 1994). Seed germination characteristics will strongly influence the latitudinal gradient dispersal in China. In general, *S*. *alterniflora* has no persistent seed bank because seed survival time is less than a year. They remain dormant until the following spring after they mature (Wang et al., 2006). The adaptability of germination to the environment is one of the key driving factors affecting distribution (Liu et al., 2021).

### Seed Collection and Cold Stratification

**Figure 1:**
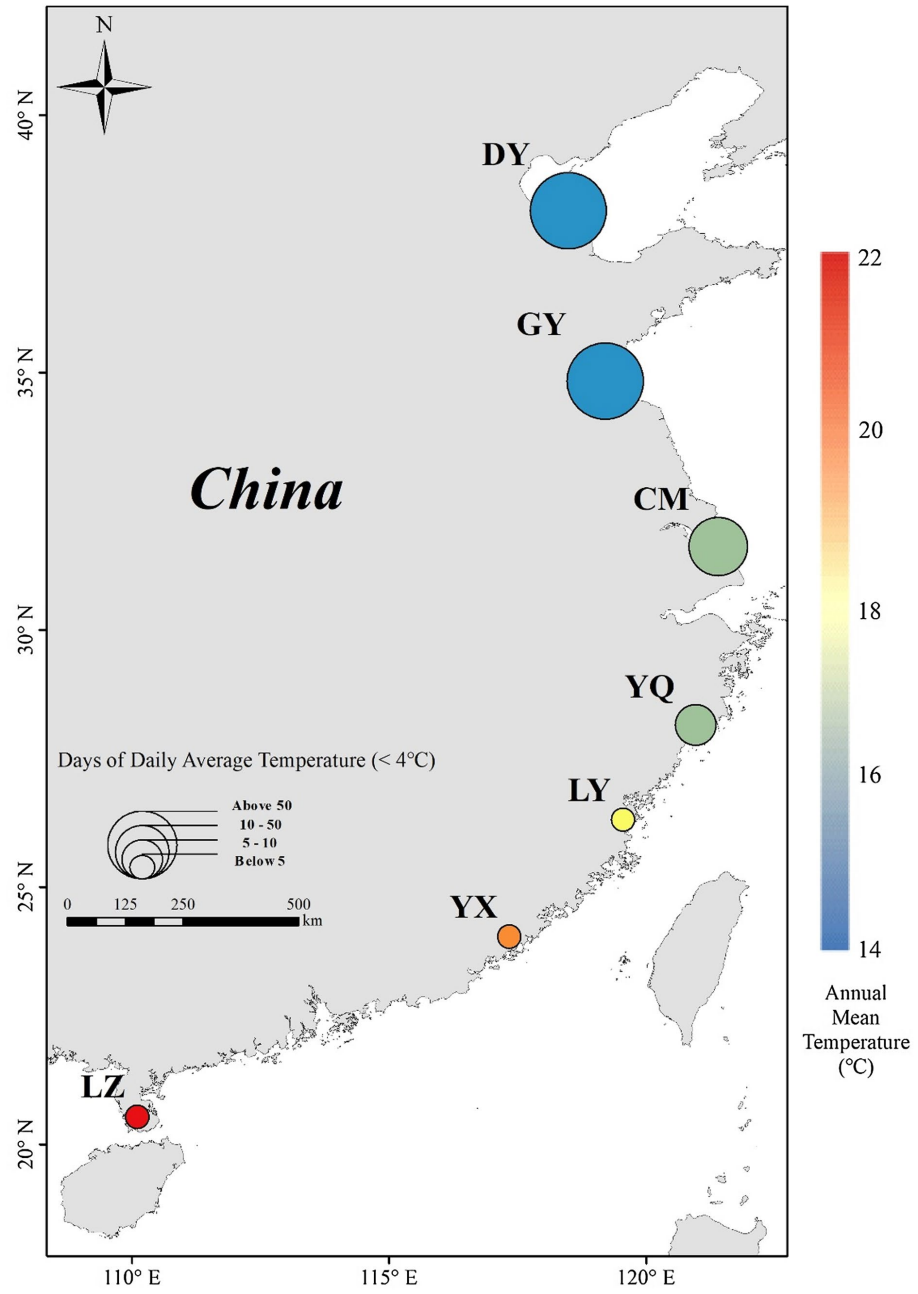
*Spartina alterniflora* seed collection locations in China (circle), and annual mean temperature (indicated by color) and days of average temperature < 4°C (indicated by size of circle) variation across latitude. See Table 1 for site code.

In September–November, 2020, we collected *S. alterniflora* seeds across a wide latitudinal gradient from the introduced range in China. We sampled seven locations between 38.15°N and 20.54°N (Figure 1; Table 1). According to the climate data (2009–2019) from China Meteorological Data Service Center,^1^ annual mean temperature at the locations varied from 14.1°C to 23.5°C, and annual maximum and minimum temperature varied from 10.0°C to 27.1°C. To explore seeds’ response to the nature temperature range and below or up the nature temperature range, we set up 4 temperature treatments of 5°C, 15°C, 25°C, or 35°C. And, days of daily average temperature below 4°C indicates the seeds cold stratification time (Biber and Caldwell, 2008; Wu et al., 2019). It can be seen that seeds under natural treatments go through 0 to over 3 months of temperature below 4°C. Therefore, we set up four treatments of 0/1/3/5-month cold stratification time.

**Table 1:** Geographic locations and climate (annual mean temperature, annual maximum temperature, annual minimum temperature, and days of daily average temperature below 4°C of seven survey locations on the coast of China) of seven populations of *Spartina alterniflora* were used in this study.

Provenance	Abbreviation	Latitude (°N)	Annual mean temperature (°C)	Annual maximum temperature (°C)	Annual minimum temperature (°C)	Days of daily average temperature below 4°C
Dongying	DY	38.15	14.1 ± 0.2	19.4 ± 0.2	10.0 ± 0.1	94.5 ± 0.7
Ganyu	GY	34.84	14.3 ± 0.2	18.8 ± 0.2	10.2 ± 0.2	79.7 ± 1.0
Chongming	CM	31.62	17.4 ± 0.1	21.1 ± 0.1	14.2 ± 0.1	26.4 ± 0.8
Yueqing	YQ	28.15	17.9 ± 0.1	20.9 ± 0.1	15.8 ± 0.1	9.6 ± 0.7
Luoyuan	LY	26.31	20.3 ± 0.1	24.0 ± 0.2	17.6 ± 0.1	0.4 ± 0.1
Yunxiao	YX	24.04	21.4 ± 0.1	25.5 ± 0.1	18.8 ± 0.1	0.1 ± 0.0
Leizhou	LZ	20.54	23.5 ± 0.2	27.1 ± 0.2	21.0 ± 0.2	0.1 ± 0.0

Locations are ordered from north to south. ±1 indicates SE.

Within each location we worked at two sites, separated by no <2 km (Liu et al., 2016; Figure 1). At each site, we established five randomly positioned quadrats of 0.5 m × 0.5 m. Within each quadrat, we randomly collected whole inflorescences from 10 individual plants. Previous studies have shown that filled seeds contain an embryo and endosperm, and can potentially germinate and grow; unfilled seeds have neither of these issues and cannot germinate or grow (Daehler and Strong, 1994; Ayres et al., 2007). We hand-sorted filled seeds from unfilled seeds and counted them (Daehler and Strong, 1994; Liu et al., 2016). Each provenance is with 10 samples, each sample contained more than 400 filled seeds. Before the germination experiments, the seeds were cleaned with ultra-pure water, cover with 10‰ seawater, placed in sealed and labeled plastic bags within 1 month of collection, and then stored in a refrigerator at 4°C for a 0-, 1-, 3-, or 5-month stratification period to break their dormancy (Plyler and Carrick, 1993; Koning, 2006). We removed the seeds that germinated before the germination experiment.

### Germination Experiment

To monitor germination, seeds were placed into Petri dishes containing 1 sheet of filter paper and wetted with ultra-pure water to keep seeds moist. The filter paper was wetted or misted daily to prevent seeds from any drying that would cause mortality (Plyler and Carrick, 1993). Dishes were placed in a growth chamber under one of four temperature regimes: 5°C, 15°C, 25°C, or 35°C. Over the first 10 days, the number of germinated seeds was recorded daily. From day 11 to 98, observations were made every 2 days. The seed germination and flowering phenology varied across latitude in the field (Liu et al., 2017a; Qiu et al., 2018; Chen et al., 2021), so the stratification/storage time between seed maturation and germination is different. Samples that did not undergo stratification were placed to germinate in December. Those that experienced 1 month of stratification were placed to germinate at the end of January, those with 3 months of stratification at the end of March, and those with 5 months of stratification at the end of May. Each trial was followed for 98 days, by which time the most of seeds had reached the germination plateau. The final plateau was determined when no germination had occurred for 5 successive days for most of the dishes (Grime et al., 1981). In total, therefore, 16 germination trials were run for each location (four stratification treatments × four temperature treatments) with 20 seeds in each.

To compare germination among treatments and location, we calculated the germination rate (G%), germination index (GI), median germination time (T_50_), and mean germination time (MGT). Germination rate reflects seed activity, germination index reflects seed germination vigor, mean germination time and T_50_ reflect seed germination speed, and the four indices jointly reflect the germination potential of *S. alterniflora* seeds under the action of different environmental factors. The cumulative germination rate (G%) of *S. alterniflora* seeds was calculated as follows:


$$G\%=\frac{n}{N}*100\%$$

where *n* is the cumulative germination number at a specified time and *N* is the total number of tested seeds (Su et al., 2011; Yang et al., 2020).

The germination index (GI) was calculated as follows:


$$\mathrm{GI}=\overset{98}{\underset{i=1}{\sum }}\left(G_{t}/D_{t}\right)$$

where G*_t_* is the number of germinations at different times and D*_t_* is the corresponding number of germination days (Zhou et al., 2021).

The median germination time (T_50_) was the time for 50% of final germination (Ranal and Santana, 2006).

The average germination time (MGT) was calculated as follows:


$$\mathrm{MGT}=\overset{t}{\underset{i=1}{\sum }}N_{i}D_{i}/\overset{t}{\underset{i=1}{\sum }}N_{i}$$

where N_i_ is the number of seeds germinating on day i, D_i_ is the number of germination days (Ranal and Santana, 2006).

### Statistical Analysis

To confirm the effects of temperature, cold stratification, and their interaction on germination traits, we used a mixed model with temperature, cold stratification, and their interaction as fixed factors, and with latitude (provenance) as random effects. To confirm the differences in latitudinal clines under different temperatures, we used general linear models, and the two-way ANOVA with latitude and temperature as main factors, to determine the main and interacting effects on germination traits under each cold stratification treatment. Data were cube-root (*x*)-transformed or cube-root [ln(*x* + 0.1)]-transformed or cube-root (*x*/10 + 1)-transformed or arcsine [cube-root (x/100)]-transformed to improve the normality of errors and homogeneity of variance when necessary. To explore germination variation across latitude, we used linear regressions to analyze the relationships between germination traits (germination rate, germination index, T_50_ and mean germination time) and latitude of origin. To identify the gradients in climate, a principal component analysis (PCA) was conducted with rda function in the vegan package (Oksanen et al., 2019). To determine the latitude effect variation under different treatments, we did the linear regressions between the slopes of germination traits vary by latitude under different cold stratification and temperature. We performed all analyses in R version 4.1.2 (R Core Team, 2021).

## Results

### Cumulative Germination Varied by Stratification Time and Temperature

**Figure 2:**
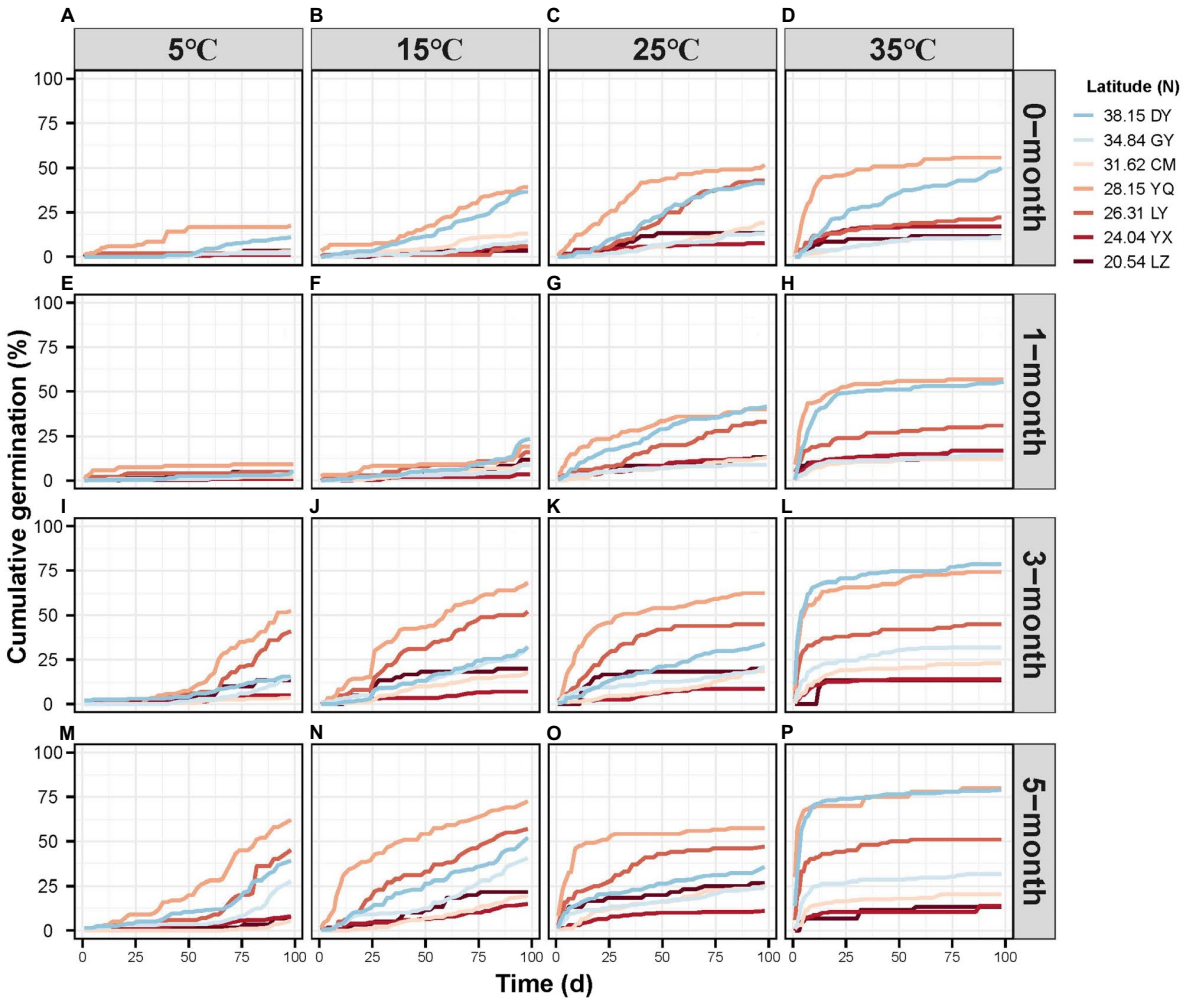
The cumulative germination percentage per observation day of different latitude sites under different stratification time (**A-D** 0-month; **E-H** 1-month; **I-L** 3-month; M-P 5-month) and temperature (**A, E, I, M** 5 °C; **B, F, J, N** 15 °C; **C, G, K**, **O** 25 °C; **D, H, L, P** 35 °C).

Cumulative germination percentage varied considerably under different treatment conditions (Figure 2). Germination rate at 98 days ranged from <10% with 1-month stratification at 5°C (Figure 2E) to >75% germination with a 5-month stratification at 35°C (Figure 2 P). Seeds reached germination plateau most rapidly at 35°C and 5-month stratification treatment (Figure 2P). As we predicted, in general, with longer stratification time and at the higher temperature, germination occurred earlier and was more rapid. Even so, germination was also determined by latitude. Seeds from lower latitudes tended to have lower and slower germination rates.

### Germination Traits Response to Stratification Time and Temperature

**Figure 3:**
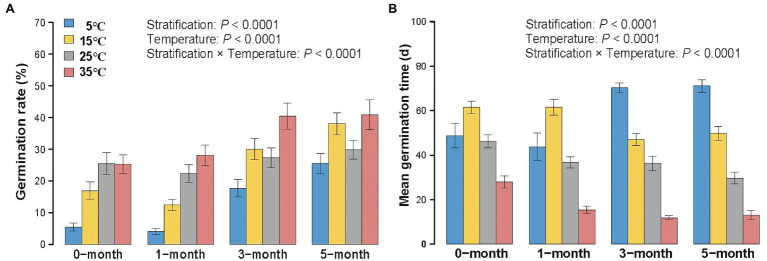
Variation of germination rate **(A)** and mean germination time **(B)** of 0/1/3/5-month-stratification *Spartina alterniflora* seeds under different temperatures (5°C–35°C). Values indicate means ± 1 SE.

In line with our predictions, longer stratification time and higher temperature both increased mean germination rate (Chisq=158.6,P<0.0001 for stratification treatments; Chisq=182.31,P<0.0001 for temperature treatments) and germination index (Chisq=68.29,P<0.0001 for stratification treatments; Chisq=63.32,P<0.0001 for temperature treatments; Figure 3A; Supplementary Figure S1A). Meanwhile, longer stratification and higher temperatures tended to decrease both mean germination time (Chisq=26.37,P<0.0001 for stratification treatments; Chisq=684.76,P<0.0001 for temperature treatments) and T_50_ (Chisq=24.14,P<0.0001 for stratification treatments; Chisq=559.27,P<0.0001 for temperature treatments; Figure 3B; Supplementary Figure S1B). Moreover, for germination rate, mean germination time, germination index and T_50_, there was a significant interaction between stratification and temperature (Chisq=35.82,P<0.0001 for germination rate; Chisq=98.78,P<0.0001 for mean germination time; Chisq=19.84,P=0.02 for germination index; Chisq=96.94,P<0.0001 for T_50_; Table 2).

**Table 2:** Mixed model analysis of germination rate, mean germination time, germination index and T_50_ of *Spartina alterniflora*, cold stratification and temperature as fixed effect with latitude (provenance) as random effect.

Factor	Germination rate			Mean germination time			Germination index			T_50_		
	Chisq	*Df*	*P*	Chisq	*Df*	*P*	Chisq	*Df*	*P*	Chisq	*Df*	*P*
Stratification (S)	158.60	3	**<0.0001**	26.37	3	**<0.0001**	68.29	3	**<0.0001**	24.14	3	**<0.0001**
Temperature (T)	182.31	3	**<0.0001**	684.76	3	**<0.0001**	63.32	3	**<0.0001**	559.27	3	**<0.0001**
S * T	35.82	9	**<0.0001**	98.78	9	**<0.0001**	19.84	9	**0.02**	96.94	9	**<0.0001**

The significant p values were shown in bold (p < 0.05).

### Germination Variation Across Latitude Relating to Stratification Time and Temperature

**Figure 4:**
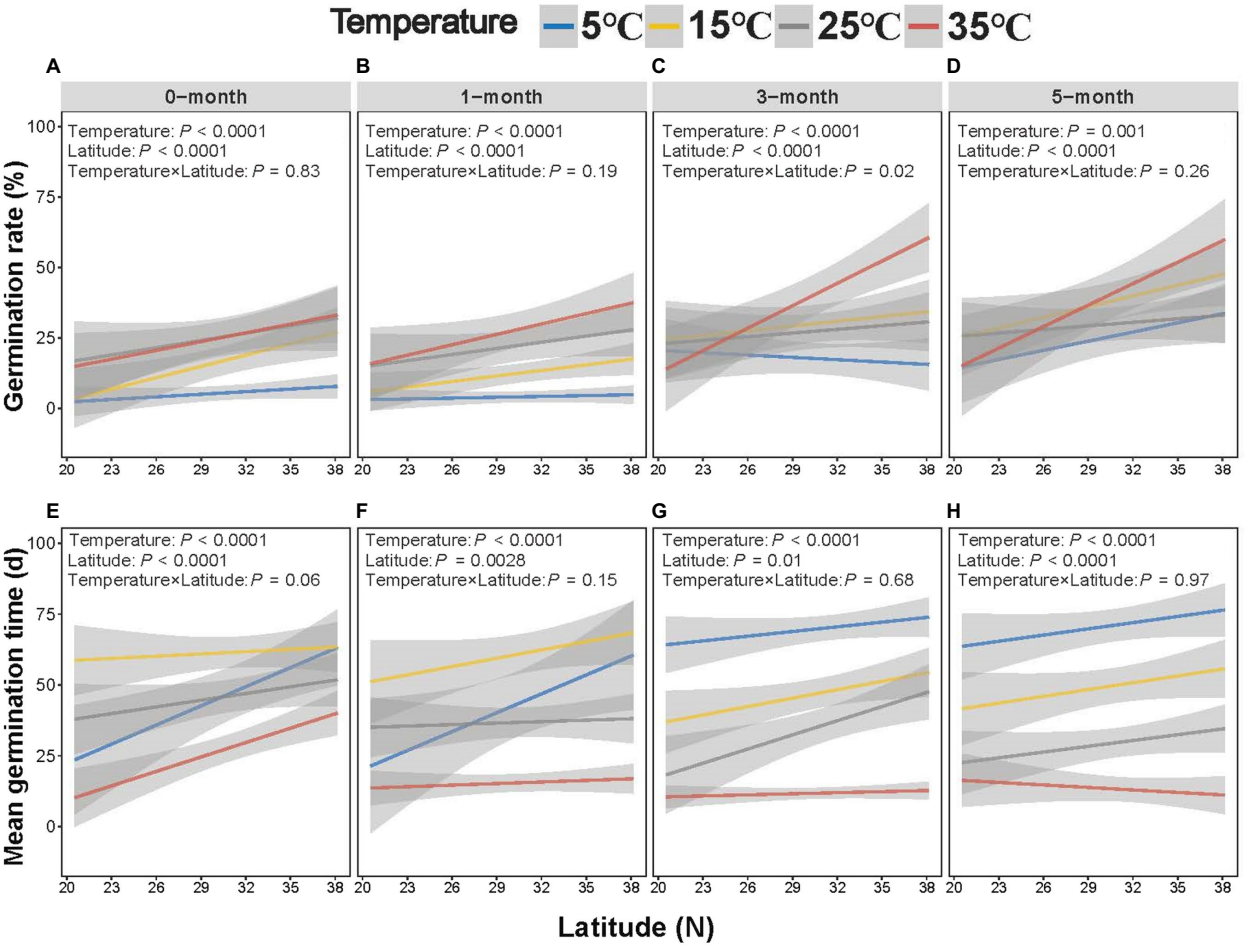
Effect of temperature/stratification treatments on the germination rate **(A–D)** and mean germination time **(E–H)** of *Spartina alterniflora* seeds from different latitudinal provenances (shaded area indicates 95% CI).

**Figure 5:**
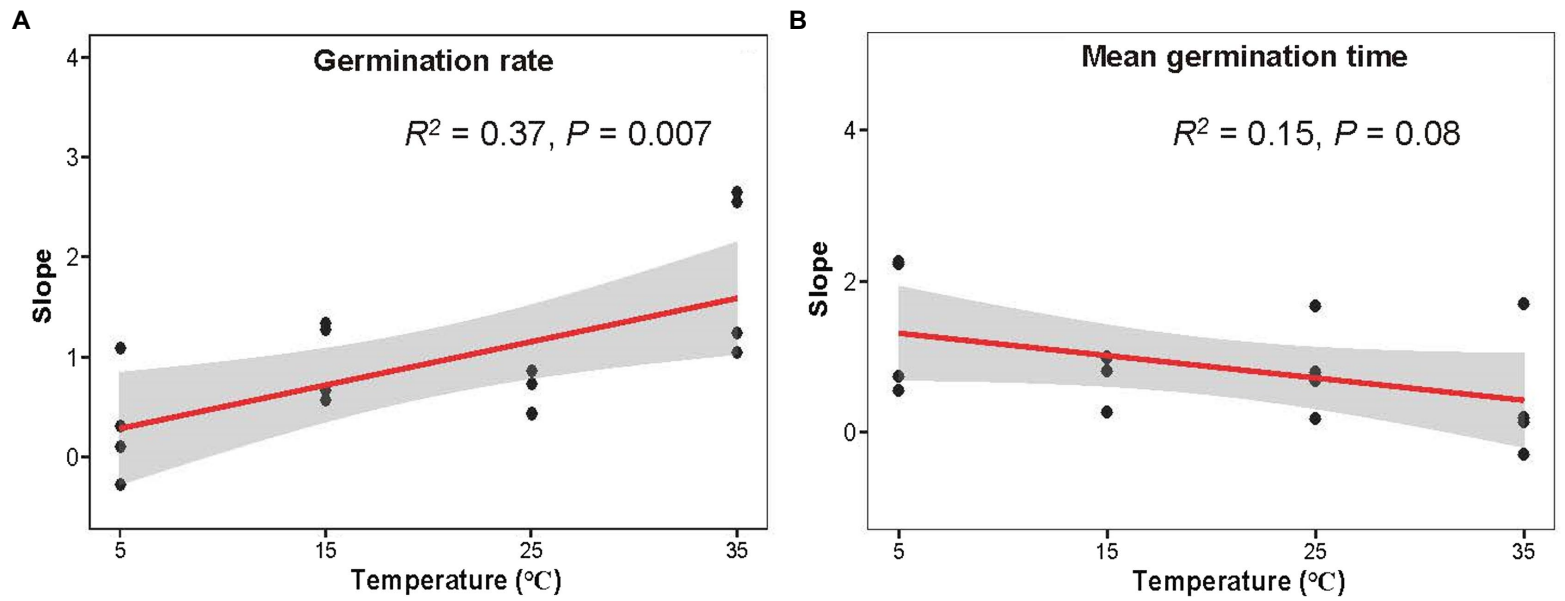
Variation of germination rate **(A)** and mean germination time slopes **(B)** of latitude influence of 0/1/3/5-month-stratification *Spartina alterniflora* seeds under different temperatures (shaded area indicates 95% CI). Slope was from Figure 4.

Germination rate increased with latitude, which interacted with stratification time and temperature (Figures 4A–D). It can be seen that the slope of germination rate and germination index to latitude increased with temperature, while germination time decreased (Figure 5; Supplementary Figure S3). Seeds exposed to the higher temperature and longer stratification had higher germination rates than seeds that were exposed to shorter stratification times and lower temperatures, and the effects of this were greater in seeds from higher latitudes due to the higher slope of germination rate to latitude (Figure 5A). A similar interaction was observed for germination index (Supplementary Figures S2C,D, S3A), while opposite for T_50_ and germination time with decreasing trends (Figure 5B, Supplementary Figure S3B). Seeds exposed to colder temperatures took longer to germinate than those exposed to warmer temperatures, but those from lower latitudes were faster to germinate than seeds from higher latitudes at the coldest temperature at short stratification times (Figures 4E–H, 5B; Supplementary Figures S2E-H, S3B). The combination of latitude, cold stratification and the temperature contributed to the final germination. Principal component analysis of the five climate variables across latitude that the first PC axes explained almost all variation in climate gradients (94.67%) and were mainly associated with annual min temperature and days of daily average temperature below 4°C (Supplementary Figure S4).

## Discussion

Experimental exposure to cold stratification and temperature treatments affected the germination traits of *S. alterniflora* seeds of different provenances along a latitudinal gradient in China. Prolonging stratification time and higher temperature both promoted seed germination rate and shortened seed germination time. Moreover, these two factors interacted with each other to influence the germination characteristics of seeds. We found that germination rate and index increased linearly with latitude, but germination time decreased with latitude. Cold stratification interacted with temperature to influence this relationship. With the greatest slope in five-month stratification, lower slope in one or three-month stratification, and no relationship in zero stratification, germination rate shows the latitude effect. And with higher temperature and longer stratification time, the latitude effect becomes more significant. The adaptability of *S. alterniflora* seed germination to temperature and cold stratification across latitude is different, which reflects the ecological adaptability of *S. alterniflora* to the coastal ecosystem and its invasion potential to large-scale latitude in China. With climate change and global warming, it may therefore have the potential of evolution. This study predicts global warming is likely to accelerate the establishment, naturalization, and spread of invasive plants of *S. alterniflora* in higher latitudes in China.

### Effects of Cold Stratification on Seed Germination

Cold and wet storage (stratification) is essential to break dormancy and promote the germination of seeds of *S. alterniflora*. A minimum of 4–8 weeks at 4°C appears necessary (Biber and Caldwell, 2008). Our findings confirm that cold stratification is an effective agent for breaking the seed dormancy and improving germination in *S. alterniflora*. The importance of cold stratification has been confirmed in many species, including *Primula beesiana* (Yang et al., 2020), *Aster tripolium*, and *Triglochin maritimum* (Al-Hawija et al., 2012), and *Guizotia scabra*, *Parthenium hysterophorus*, *and Verbesina encelioides* (Karlsson et al., 2008); while a moderate thermal stratification has a positive influence on seed germination in *Cirsium arvense* (Bochenek et al., 2009). This requirement for cold stratification also helps to prevent winter germination.

Cold stratification appears to have several benefits. First, it can improve superoxide dismutase (SOD) activity in seeds, removing any accumulated O_2_^−^, an adaptation of seeds to the low-temperature environment in winter. Second, levels of abscisic acid (which inhibits germination) decrease significantly during cold stratification, coincident with an increase in germination capacity (Feurtado et al., 2004). In addition, cold stratification can also break seed dormancy in halophytes (Kołodziejek and Patykowski, 2015) and allow germination at the right time to escape direct salt stress. For example, *S. alterniflora* seeds from North Carolina, United States, have a dormancy period of 6–12 months (Stalter, 1973) and germination of invasive *Spartina* in China coincides with spring rains and rises in temperature (Yuan and Shi, 2009). In contrast to previous studies (Stalter, 1973; Seneca, 1974), we found no negative effect of a stratification period over 4 months, but that germination rate and index were even greater at 5 months. This difference may be because our data are from invasive populations, which may be different from native populations. Stratification shortened T_50_ and MGT above 15°C but showed the opposite pattern at 5°C. Further research is needed to support and analyze this condition.

### Effects of Temperature on Seed Germination

The role of temperature in regulating the seed dormancy of halophytes is known for some species (Ajmal Khan et al., 2000), such as *Halogeton glomeratus*, *Lepidium latifolium*, and *Peganum harmala* (Ahmed and Khan, 2010), and *Puccinellia nuttalliana* and *Puccinellia distans* (Tarasoff et al., 2007). *S. alterniflora* is also known to be sensitive to changes in temperature (Kirwan et al., 2009). The invasive spread of *S. alterniflora* is closely related to local temperature (Loebl et al., 2006; Liu et al., 2016). It was found that number of shoots which indicated clonal growth increased slightly with temperature (Liu et al., 2016). Our results show that germination responds positively to higher temperatures, with or without a period of cold stratification, consistent with previous studies (Zhu et al., 2011; Hayasaka et al., 2020). In future climate scenarios, the increase with temperature on both clonal growth and sexual reproduction may lead to population expansion.

Seeds undergo active metabolic reactions during germination. As such, within a certain range, germination processes are accelerated as temperature increases (Zhang et al., 2012), but too low or too high a temperature will affect germination due to membrane permeability, membrane-binding activity, and enzyme denaturation (Gul and Weber, 1999; Gulzar et al., 2001). The optimal temperature for seed germination is closely related to the original habitat of the population (Zhang et al., 2012). Our experimental temperature treatments (5°C–35°C) coincided with the suitable range experienced in nature, and the field temperatures experienced by *S. alterniflora* are rarely (if ever) more than 35°C (Table 1; Figure 1). In subsequent experiments, we can set higher temperature treatments. Temperature cues are particularly important for the release of dormancy to allow germination and may be particularly affected by rising temperatures due to climate change (Loarie et al., 2008; Footitt et al., 2013, 2018; Baskin and Baskin, 2014). Also, it is noteworthy that cold days is important which can delay the germination (Milbau et al., 2009; Chamorro et al., 2017; Gremer et al., 2020).

### Latitudinal Variation in Seed Germination of *Spartina alterniflora*

Our results show an apparent increase in germination rate in seeds from higher latitudes, consistent with the cross-species analysis of herbs by Graae et al. (2009). Moreover, our results demonstrate that germination traits of seeds of *S. alterniflora* from different provenances at different latitudes differ significantly in response to stratification and ambient temperature. This effect of latitude was especially strong in terms of germination index with higher temperatures (Chamorro et al., 2018; Wang et al., 2021), which indicates that seed germination of populations at higher latitude was more sensitive to temperatures (Cochrane et al., 2015). Therefore, population recruitment at higher latitude may benefit from warmer climate accelerates germination (Wu et al., 2019). The responses of seeds from different latitudes to stratification were significantly different (Fowler and Dwight, 1964). This is because low-latitude populations lack a cold stratification requirement, and high-latitude populations experience cold temperatures and need cold stratification to release dormancy (Weng et al., 2006; Cortés-Fernández et al., 2021). Reproductive traits, such as seed set and seed survival of *S. alterniflora* also increase with latitude (Liu et al., 2017a). Invasive plant populations are likely very plastic and can adapt to different local environments (Feng et al., 2009; Liu et al., 2017a), and their traits will co-vary with these conditions that vary with latitude and altitude (Colautti et al., 2009; Castillo et al., 2014). Although it is difficult to determine the balance of genetic vs. environmental influence (Weng et al., 2006; Cortés-Fernández et al., 2021), our results indicate that *S. alterniflora*, which is distributed over a wide latitudinal range, may have significant adaptive variation in seed germination.

With global warming, this variation may lead to a mismatch between the optimal germination time of *S. alterniflora* seeds and the environmental cues of previous adaptation (Walck et al., 2011; Cochrane, 2019; Sentinella et al., 2020). These variations in space and time suggest that a warmer climate may improve the long-term survival of *S. alterniflora*, as well as its competitive abilities (Kirwan et al., 2009; Clements and Ditommaso, 2011). High-latitude provenances have existing high adaptability to a cold environment (Mao et al., 2015). Climate warming may affect the population dynamics of species by altering seed germination patterns, especially for frontier populations near the boundaries of their distribution (Wu et al., 2019), which should be a focus of future research.

### Interaction Effects

Cold stratification requirements are especially common in species from high latitudes (Wagner and Simons, 2008), as our results demonstrated. Previous studies have found that seeds from high-latitude populations that experience more severe climates have deeper dormancies that require longer periods of cold stratification to overcome (Cavieres and Arroyo, 2000, 2001; Bernareggi et al., 2016). In contrast, low-latitude populations lack a cold stratification requirement and experience temperatures conducive for germination (Graae et al., 2009). Our finding that cold stratification improves seed germination from high latitudes is consistent with the results of previous work on arctic and alpine species (Cavieres and Arroyo, 2000; Liu et al., 2017b), which all experience a severe abiotic environment.

Local climate will differentially affect populations of *S. alterniflora* across its invasive range (Liu et al., 2017a). Seeds of populations from cooler climates (high-latitude sites) could germinate earlier under warmer temperatures as a result of the earlier onset of the warm season or regional warming (Picciau et al., 2019). In a climate change context, this response would favor a potential expansion toward northern or higher elevation sites. On the other hand, similar changes to local climates would negatively affect germination in lower latitude sites, which may lead to a range reduction at the warmer end of the distribution. Within the temperature gradient, we set up, germination of *S. alterniflora* was always promoted, showing that the existing invasion region of *S. alterniflora* is a suitable range of temperature for it. Future work could examine germination potential at even higher temperatures (including soil temperature) and conduct field observations and real reciprocal experiments in the field.

Our results show that with longer stratification time, temperature plays a more important role in seed germination. Stratification and temperature, alongside latitude, are thus important drivers of population and community dynamics (Huang et al., 2016), by their influence on plant fitness, habitat selection, or niche construction (Donohue et al., 2005, 2010). Including more than one variable to more fully capture the germination process can facilitate an understanding of how local climate characteristics can affect germination across the distribution of a species. The germination responses of the seeds we tested provide insights into possible future effects of climate change on *S. alterniflora* distribution. This study suggests that global warming may accelerate the expansion and spread of invasive *S. alterniflora* in higher latitude coastal environments of China.

## Data Availability Statement

The data presented in the study are deposited in the Dryad repository at https://doi.org/10.5061/dryad.rbnzs7hdd.

## Author Contributions

WL, YZ, and JC conceived and designed the research. JC, YZ, WH, XW, and WL performed the experiments. HH, JC, and WL analyzed the data. JC and WL wrote the manuscript. WL and YZ provided the funding. All authors contributed to the article and approved the submitted version.

## Funding

This study was supported by the National Natural Science Foundation of China (grant nos. 32001234, 32025026, and 31971500), the Fundamental Research Funds for the Central Universities of China (grant no. 20720210075), and XMU Training Program of Innovation and Enterpreneurship for Undergraduates (grant no. 202010384136).

## Conflict of Interest

The authors declare that the research was conducted in the absence of any commercial or financial relationships that could be construed as a potential conflict of interest.

## Publisher’s Note

All claims expressed in this article are solely those of the authors and do not necessarily represent those of their affiliated organizations, or those of the publisher, the editors and the reviewers. Any product that may be evaluated in this article, or claim that may be made by its manufacturer, is not guaranteed or endorsed by the publisher.

## Footnotes

^1^ http://data.cma.cn
