# Supplementary material for: Unraveling the Effects of Cold Stratification and Temperature on the Seed Germination of Invasive Spartina alterniflora Across Latitude
Source: Front Plant Sci. 2022 Jun 29;13:911804. doi: 10.3389/fpls.2022.911804 (PMC9277449; doi:10.3389/fpls.2022.911804)
Supplement: Supplementary file 1 [file Data_Sheet_1.docx]

**Supplementary Figure Captions**

**Fig. S1** Variation of germination index (A) and T_50_ (B) of 0/1/3/5-month-stratification *Sparitna* *alterniflora* seeds under different temperatures (5-35^o^C). Values indicate means ± 1 SE.

**Fig. S2.** Effect of temperature/stratification treatments on the germination index (A-D) and T_50_ (E-H) of *Sparitna* *alterniflora* seeds from different latitudinal provenances (Shaded area indicates 95% confidence intervals).

**Fig. S3** Variation of germination index (A) and T_50_ slopes (B) of latitude influence of 0/1/3/5-month-stratification *Spartina* *alterniflora* seeds under different temperatures.

**Fig. S4** Principle components analysis of climate factors of each site.

**Fig. S1**


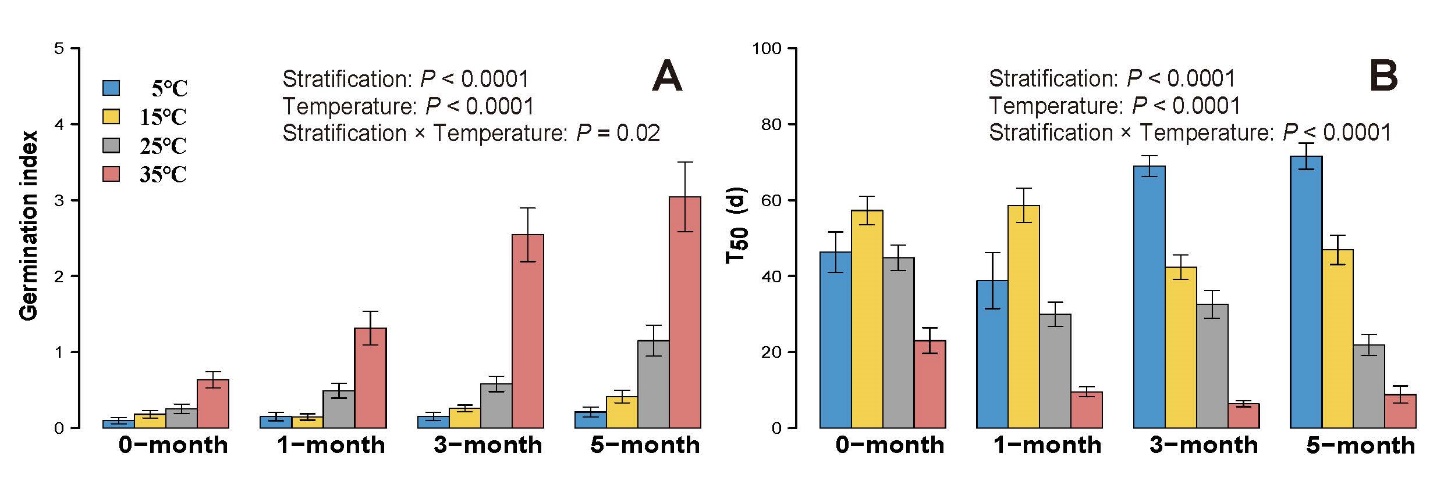


**Fig. S2**


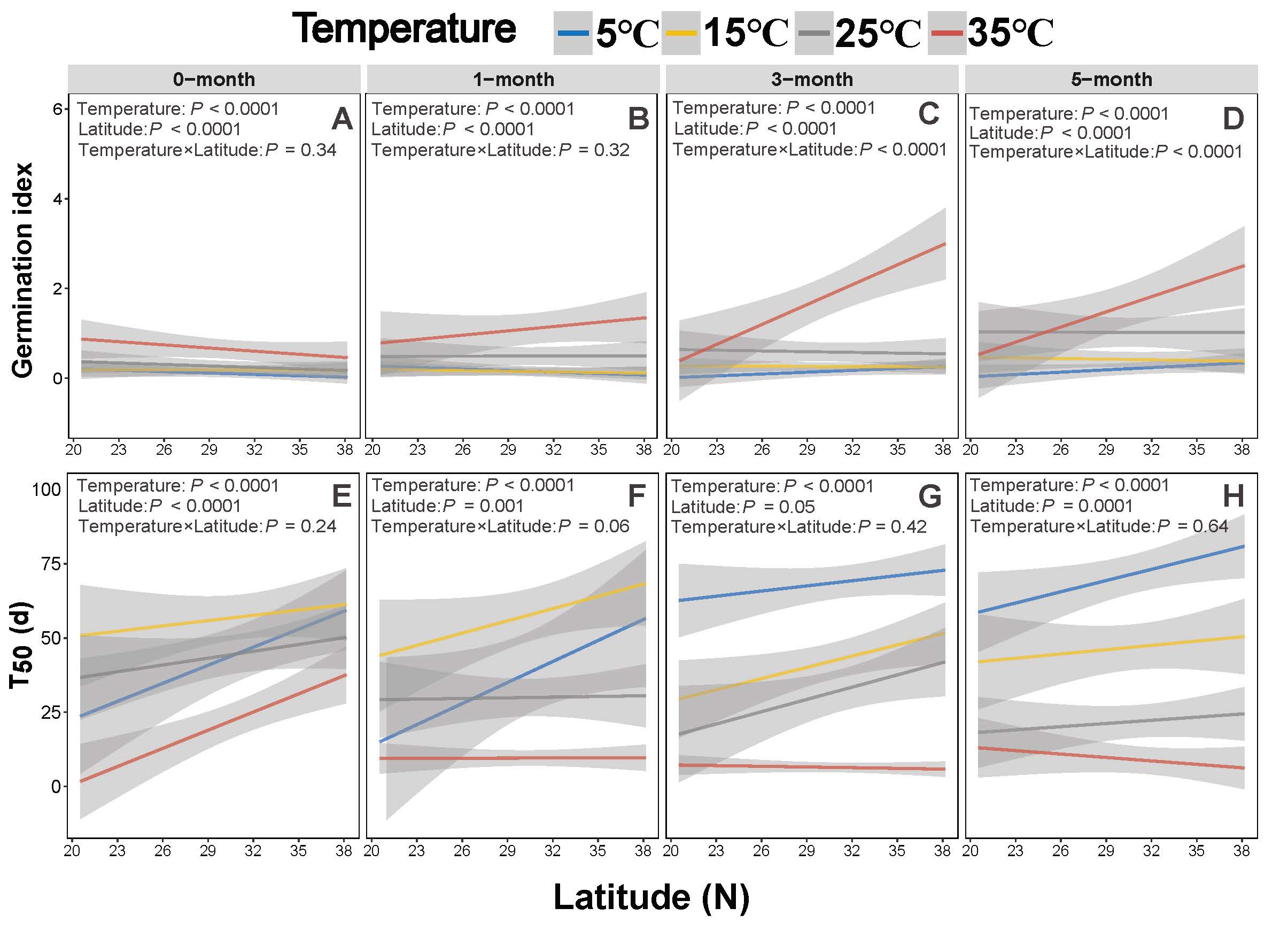


**Fig. S3**


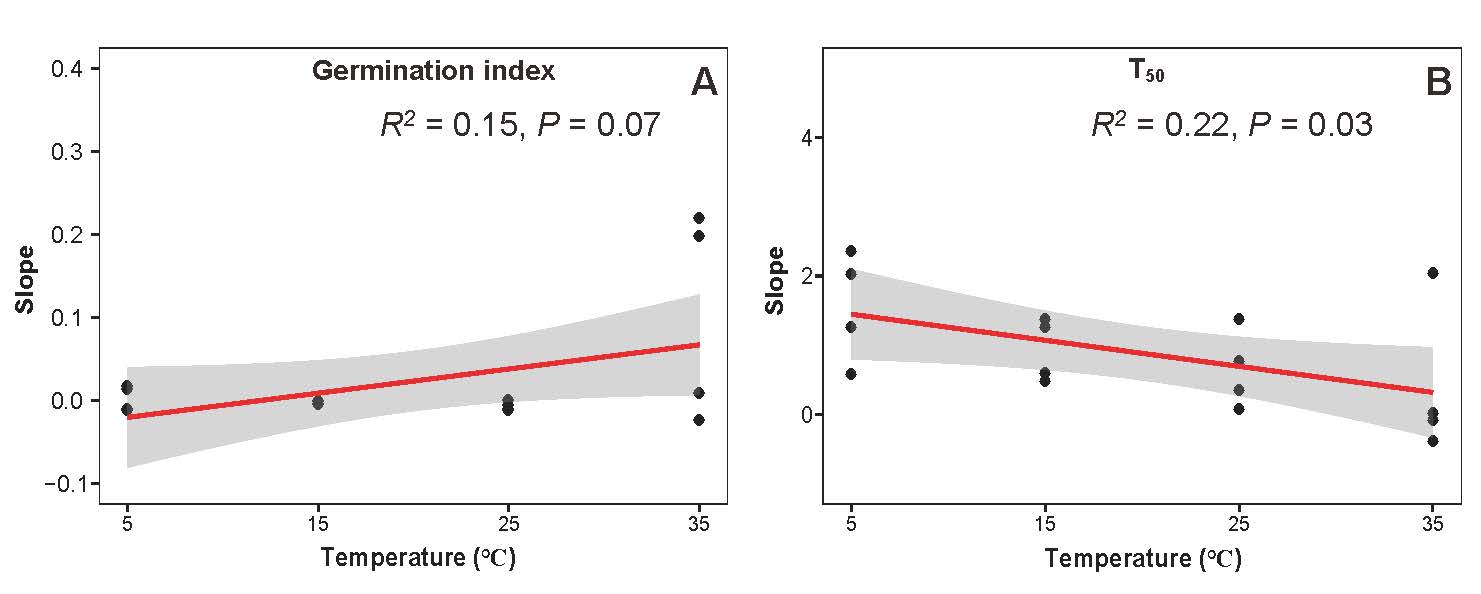


**Fig. S4**

*
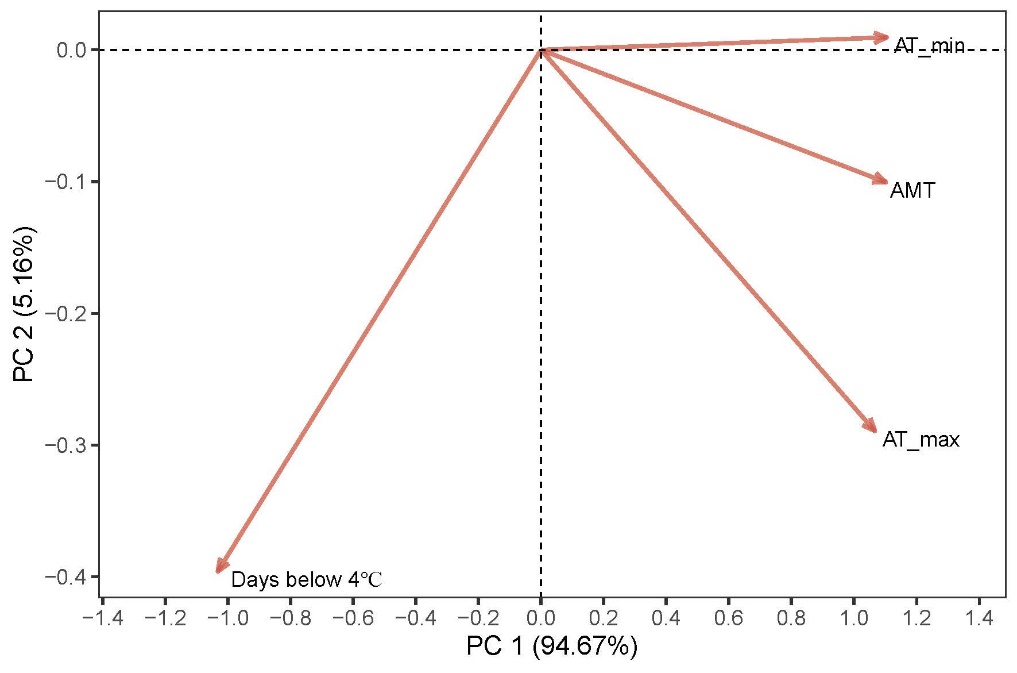
*
